# The complete chloroplast genome sequence of *Clematis Montana* Buch.-Ham. (Ranunculaceae) and its phylogenetic analysis

**DOI:** 10.1080/23802359.2020.1771225

**Published:** 2020-06-01

**Authors:** Chaoyi Mao, Xueting Zhang, Jia Shi, Sha Chen

**Affiliations:** Institute of Chinese Materia Medica, China Academy of Chinese Medical Sciences, Dongzhimennei, Beijing, China

**Keywords:** *Clematis montana*, chloroplast, Illumina sequencing, phylogeny

## Abstract

*Clematis montana* is a medicinal plant commonly used in southwest of China. The complete chloroplast (cp) genome sequence of *C. montana* was sequenced using the Illumina Hiseq 4000 platform. The cp genome of *C. montana* was 159,523 bp in length with 37.98% overall GC content. This circular molecule had a typical quadripartite structure containing a large single-copy (LSC) region of 79,385 bp, a small single-copy (SSC) region of 18,092 bp, and two inverted repeat (IR) regions of 31,023 bp. The cp genome contained 135 genes, including 91 protein-coding genes, 36 tRNA genes, and 8 rRNA genes. Phylogenetic analysis based on whole cp genome sequences showed that *C. montana* was closest to *C. alternata.*

*Clematis montana* Buch.-Ham. belongs to the family of Ranunculaceae, which woody climber distributed in temperate Himalaya up to an altitude of 4000 m (Singhal et al. 2010). The dry stem of *C. montana* was used as traditional Chinese Medicine for the treatment of inflammatory conditions, such as rheumatism, urinary tract infection, and so on (Chawla et al. 2012; Bhatt et al. 2016). Previous studies mainly focused on describing its chemical compositions and their pharmacological activities. In this study, we reported the complete cp genome and phylogenetic analysis of *C. montana* for the first time, which will contribute to further studies on its genetic research and resource utilization.

Fresh leaves of *C. montana* were sampled from the Nanjing Botanical Garden Men. Sun Yat-Sen, Institute of Botany Jiangsu Province and Chinese Academy of Sciences (Nanjing, China, N32°03′6.64″, E118°49′39.54″). The voucher specimen was deposited in the herbarium of Institute of Chinese Meteria Medica, China Academy of Chinese Medical Sciences (accession number: HBGP0230_NJ). Genomic DNA was extracted using the DNeasy plant mini kit (Qiagen). The whole-genome sequencing was conducted with 350 bp pair-end reads on the Illumina NovaSeq system (Illumina, San Diego, CA). In total, 4.02 Gb of raw data (26,813,858 reads) were obtained. *De novo* genome assembly and annotation were conducted by NOVOPlasty (Dierckxsens et al. 2017) and GeSeq (Tillich et al. 2017), respectively. The annotated cp genome was deposited in the GenBank (accession number: MT292622).

The result showed that the cp genome of *C. montana* was 159,523 bp in length, with a large single-copy region (LSC) of 79,385 bp, a small single-copy region (SSC) of 18,092 bp, and a pair of inverted repeat (IR) regions of 31,023 bp. A total of 135 genes were annotated, including 91 protein-coding genes, 36 tRNA genes, and 8 rRNA genes. The GC content of the cp genome is 37.98%. To identify the phylogenetic position of *C. montana,* alignment was performed on the 13 cp genome sequences using MAFFT v7.309 (Katoh and Standley 2013). The maximum likelihood (ML) bootstrap analysis with 1000 replicates was performed using RaxML v8.2.12 (Stamatakis 2014). The phylogenetic tree showed that *C. montana* was closely related to *C. alternata* (Figure 1). The cp genome sequence of *C. montana* in this study might provide important information on phylogenetic and evolutionary studies in Ranunculaceae.

**Figure 1.**
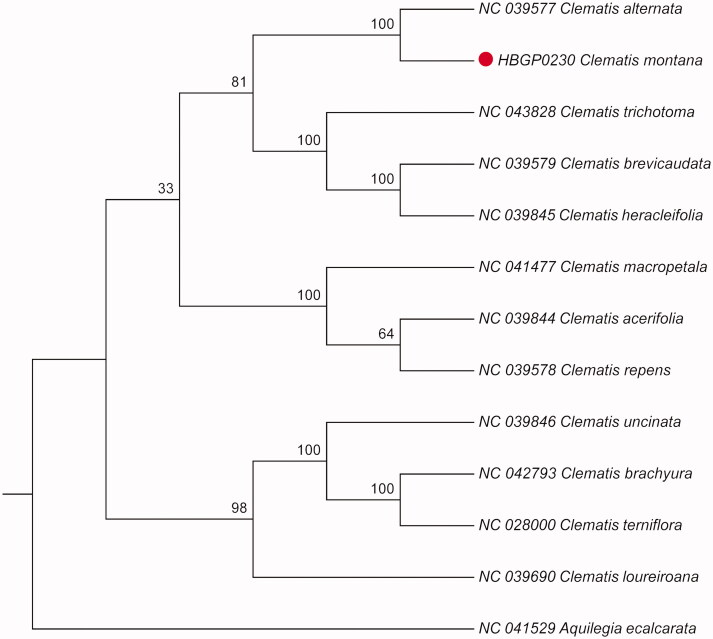
Maximum likelihood phylogenetic tree based on the complete chloroplast genome sequences of 13 plant species from Ranunculaceae.

## Data Availability

The annotated cp genome has been deposited in GenBank under accession number [MT292622]. The data that support the findings of this study are openly available in Genbank at [https://www.ncbi.nlm.nih.gov/nuccore/], reference number [MT292622].
